# Psychometric Validation and Arabic Translation of the 11-Item Circadian Type Inventory (CTI-11A) Among Shift Workers

**DOI:** 10.3390/clockssleep7040061

**Published:** 2025-10-25

**Authors:** Sara Ahmed Mansoor AlBuhmaid, Muneera Jasim Al-Rumaihi, Mohammed Adel M Albalawi, Ahmed Abdullatif Ahmed Almufarrij, Waqar Husain, Haitham Jahrami

**Affiliations:** 1 College of Medicine and Medical Sciences, Arabian Gulf University, Manama 329, Bahrain; saraamb@agu.edu.bh (S.A.M.A.); muneerajjr@agu.edu.bh (M.J.A.-R.); mohammedab@agu.edu.bh (M.A.M.A.); ahmedaam@agu.edu.bh (A.A.A.A.); 2 Department of Humanities, COMSATS University Islamabad, Islamabad Campus, Park Road, Islamabad 45550, Pakistan; drwaqar@comsats.edu.pk; 3 Government Hospitals, Manama 329, Bahrain

**Keywords:** circadian rhythm, sleep quality, shiftwork interventions

## Abstract

Circadian rhythm disruptions from shiftwork impact sleep quality and work performance, yet validated tools to assess circadian preferences in Arabic-speaking populations are scarce. This study aimed to translate and validate the 11-item Circadian Type Inventory (CTI-11) into Arabic (CTI-11A), evaluate its psychometric properties, and explore latent circadian profiles in relation to sleep quality. A cross-sectional survey in Bahrain involved 468 Arabic-speaking adults recruited via social media. The CTI-11A, assessing Languid/Vigorous (LV) and Flexible/Rigid (FR) subscales, and the Jenkins Sleep Scale (JSS) were administered. Confirmatory factor analysis (CFA), reliability tests, and latent class analysis (LCA) were conducted. Participants (mean age: 36.18 ± 10.35) showed CTI-11A total scores of 35.40 ± 6.61 and JSS scores of 5.76 ± 3.48. CFA confirmed the two-factor structure (RMSEA = 0.06, SRMR = 0.05, CFI = 0.93, TLI = 0.91), with Cronbach’s α of 0.72 (total CTI-11A). Test–retest reliability was high (ICC = 0.91). CTI-11A correlated moderately with JSS (r = 0.40, *p* < 0.001), with stronger FR-JSS (r = 0.36) than LV-JSS (r = 0.25) associations. LCA identified two classes (Class 1: 52%, vigorous/flexible; Class 2: 48%, languid/rigid), with Class 2 showing poorer sleep quality. The CTI-11A is a reliable and valid tool for assessing circadian preferences in Arabic-speaking populations, with distinct circadian profiles linked to sleep quality. While flexible/vigorous profiles associated with better sleep, languid/rigid profiles indicate higher sleep disturbance risk, informing targeted shiftwork interventions. Further refinement of the factor structure and broader regional validation are needed.

## 1. Introduction

The Circadian Type Inventory (CTI-11) [1,2,3] is a widely accepted self-report instrument developed for the purpose of accounting for individual differences with respect to the two important characteristics of human circadian rhythms: amplitude and stability. The CTI-11 has two scales: Languid/Vigorous (e.g., reflecting rhythm amplitude) and Flexible/Rigid (e.g., reflecting rhythm stability). These are endogenous characteristics that individuals vary on and are thought to be important components in an individual’s capacity to adjust to irregular work schedules (such as shiftwork). Vigorous types are more alert, and their sleep needs are reported to be less, while flexible types are considered to have more adjustment potential to irregular work schedules, particularly in the afternoon and evening.

Shiftwork represents a continual disruption to circadian rhythms, which are set by an internal oscillator that resists change, and can represent a significant source of disturbance to the circadian system. Thus, self-report measures such as the CTI are useful for indirectly assessing characteristics of an individual’s circadian rhythm in order to guide the selection of and provide counselling to shiftworkers [4,5,6].

Recent studies from 2020 to 2025 have further illuminated the cross-cultural challenges in validating circadian and sleep assessment tools, emphasizing the need for context-specific adaptations to capture diverse chronotypes and their health implications. For instance, Kang and colleagues identified four latent circadian profiles among Chinese nurses using the CTI-11, revealing that languid types exhibited higher presenteeism and poorer work-related flow, underscoring the scale’s utility in shift-working populations but highlighting cultural variations in profile prevalence compared to Western samples [7]. Research on shift work has documented escalating health risks from circadian disruptions, including increased stroke incidence via vascular dysregulation [8] and coronary heart disease mediated by gut microbiota alterations [9]. Shift work was found to be significantly and independently linked to a higher likelihood of poor sleep quality among this sample of workers [10]. These findings can inform guidelines and aid in the creation of workplace health promotion interventions designed to improve sleep quality, ultimately fostering a healthier workforce. A cross-sectional study conducted in 2022 at Jimma University Medical Center, Southwest Ethiopia, assessed the prevalence of shift work sleep disorders among healthcare professionals. The study found that 35.9% of participants experienced shift work sleep disorder, with significant associations linked to factors such as working in three shifts per day, having over 11-night shifts per month, lack of naps, stress, fatigue, and substance use [11]. These findings resonate with the umbrella review by Wu and colleagues, which reported several concerning health outcomes related to shift work: (1) a relative risk (RR) of 1.23 (95% CI: 1.15–1.31) for myocardial infarction among those who ever worked shifts; (2) an RR of 1.14 (95% CI: 1.10–1.19) for diabetes mellitus incidence; (3) an RR of 1.23 (95% CI: 1.08–1.41) for prostate cancer incidence; and (4) a critical assessment of methodological quality, with 6 out of 8 systematic reviews rated as critically low according to the AMSTAR 2 criteria [12]. The convergence of these findings emphasizes the importance of addressing circadian disruptions in shift-working populations across different cultural contexts and advocates for the development of tailored health strategies to mitigate the adverse effects of shift work on health, particularly in enhancing sleep quality and overall well-being.

The CTI-11 is based on a gradual refinement of previous measurements. The Circadian Type Questionnaire (CTQ) began development based on a hypothesis that workers exhibiting low amplitude and flexible rhythms would adjust better to shiftwork. The CTQ and later 15-item CTI versions did not enjoy considerable psychometric support featuring low to weak properties and low inter-item correlation coefficients. In a later study involving 18 items in the CTI that was included in the Standard Shiftwork Index (SSI), acceptable reliability was reported, but the authors described concerns about construct validity. At the time, Di Milia and colleagues reported better psychometric properties, executing a principal component analysis that yielded a two-factor 11-item model (CTI-11; 5 and 6 items). Structural equation modeling analysis confirmed the model and reported substantial improvement in psychometric traits in a sample of students over the initial 18-item version [1].

The CTI-11 has demonstrated reliability and validity in working populations, an important benchmark in developing scales like this one [1,13,14]. In a sample of working adults, the two constructs accounted for 50.04% of variance (LV = 20.24% and FR = 29.80%) and had minimum Cronbach’s alpha values of 0.72 for LV and 0.79 for FR. Confirmatory Factor Analysis (CFA) has generally provided support for a two-factor structure and an adequate level of construct validity. The CTI-11 has also been established measurement invariance across gender and work shifts in working populations, including a large sample of Chinese workers, indicating that the CTI-11 operates consistently in different groups. Concurrent validity was established based on correlations found between CTI-11 scores and the Composite Scale of Morningness (CSM), with LV scores typically showing a moderate correlation and FR scores a weak correlation. The CTI-11 scale has also been translated and validated in various languages, such as Persian, Chinese, and French, establishing the value of the CTI-11 in different contexts [1,2].

While there are robust psychometric properties, there are translations into multiple languages, and the sources outlined do not reference the translation and validation of an Arabic language version of the 11-item Circadian Type Inventory. Given the universality of shiftwork across several industries and the importance of circadian rhythms in work and health, the absence of a validated Arabic CTI-11 represents an important gap in the extant literature. Therefore, the purpose of this paper is to describe the procedures that were followed to translate and validate an Arabic Language version of the 11-item Circadian Type Inventory (the CTI-11A) in an Arabic working population. A valid, reliable, and non-invasive measure is therefore important to assess individuals’ vulnerability and susceptibility to disrupted sleep/wake patterns and develop preventative therapeutic approaches for individuals who may be at greater risk. We hypothesize that CTI-11A is reliable and valid in Arabic language.

## 2. Results

Table 1 presents descriptive statistics, skewness, and kurtosis for the study variables (N = 468). The mean age was 36.18 years (SD = 10.35, Range 19–55), with minimal skewness (0.11) and negative kurtosis (−1.14). The Languid/Vigorous (LV) subscale items (LV1–LV6) and Flexible/Rigid (FR) subscale items (FR1–FR5) of the Circadian Type Inventory (CTI-11A) had means ranging from 2.87 to 3.75, with standard deviations between 1.10 and 1.45. Skewness values were generally low (−0.66 to 0.05), and kurtosis was negative (−1.34 to −0.25), indicating slightly platykurtic distributions. Total scores for LV (M = 19.94, SD = 3.91), FR (M = 15.46, SD = 4.75), CTI-11A (M = 35.40, SD = 6.61), and Jenkins Sleep Scale (M = 5.76, SD = 3.48) showed minimal skewness and negative kurtosis, suggesting relatively normal distributions suitable for further analysis.

Table 2 summarizes the CFA of the CTI-11A, evaluating the LV and FR subscales (N = 468). Standardized factor loadings for LV items (LV1–LV6) ranged from 0.06 to 0.60; only LV6 was not significant, and for FR items (FR1–FR5) from 0.41 to 0.79, all were significant (*p* < 0.001). Residual covariances ranged from 0.38 to 1.00 (*p* < 0.001). The model showed acceptable fit (χ^2^(43) = 103.90, *p* < 0.001, RMSEA = 0.06 [90% CI: 0.04, 0.07], CFI = 0.93, TLI = 0.91, SRMR = 0.05), with information criteria indicating good model parsimony (AIC = 15,898.17, BIC = 16,039.22). The removal of LV6 from the model did not enhance its fitness.

Reliability was adequate, with Cronbach’s α and McDonald’s ω at 0.70 for CTI-11A, 0.60 for LV, and 0.77 for FR. Test–retest of CTI-11A after two weeks showed an interclass correlation of 0.91. The heterotrait–monotrait ratio (0.35) and Mardia’s coefficients (skewness: 5.50, *p* < 0.001; kurtosis: 149.06, *p* < 0.001) suggest discriminant validity and some non-normality, respectively.

The multigroup CFA assessed configural, metric, and scalar invariance across male and female groups. The configural model demonstrated acceptable fit (χ^2^(86) = 154.17, *p* < 0.001, CFI = 0.92, TLI = 0.90, RMSEA = 0.06 [90% CI: 0.04, 0.07], SRMR = 0.06), indicating a consistent factor structure across groups. The metric model, constraining factor loadings, showed improved fit (χ^2^(95) = 161.65, *p* < 0.001, CFI = 0.93, TLI = 0.91, RMSEA = 0.05, SRMR = 0.06), supporting equivalent loadings. The scalar model, with constrained intercepts, also fit well (χ^2^(104) = 172.27, *p* < 0.001, CFI = 0.92, TLI = 0.92, RMSEA = 0.05, SRMR = 0.07). Information criteria (AIC: 15,900.24–15,918.13, BIC: 16,107.66–16,200.23) decreased with added constraints, suggesting improved parsimony.

Table 3 presents the correlation matrix for the CTI-11A and JSS (N = 468). The LV subscale showed a moderate positive correlation with CTI-11A (r = 0.70, *p* < 0.001) and weaker correlations with FR (r = 0.16, *p* < 0.001) and JSS (r = 0.25, *p* < 0.001). The FR subscale was strongly correlated with CTI-11A (r = 0.81, *p* < 0.001 and moderately with JSS (r = 0.36, *p* < 0.001). The CTI-11A total score had a moderate positive correlation with JSS (r = 0.40, *p* < 0.001), indicating significant associations between circadian type and sleep quality measures.

Correlation analysis also indicated no significant association between age and the LV (r = 0.01, *p* = 0.91) or FR (r = −0.01, *p* = 0.81) subscales of the CTI-11A. However, a weak positive correlation was observed between LV and FR (r = 0.16, *p* = 0.0006), suggesting that circadian amplitude and stability are only minimally related in this sample.

Table 4 summarizes the LCA of the CTI-11A and JSS (N = 468), identifying two latent classes based on LV, FR, and JSS manifest items. The model yielded a log-likelihood of −3796.23, with fit indices indicating adequate model fit (AIC = 7822.45, BIC = 8299.53, CAIC = 8414.53, G^2^(352) = 1972.66, *p* < 0.001). Entropy was 0.64, suggesting moderate class separation. Class 1 comprised 52% of the sample, and Class 2 comprised 48%, indicating nearly equal prevalence of the two profiles.

## 3. Discussion

The psychometric validation of the Arabic version of the 11-item CTI-11A demonstrates robust properties, aligning closely with prior validations of the CTI-11 while revealing context-specific differences. The CTI-11A exhibited satisfactory internal consistency, with Cronbach’s α and McDonald’s ω of 0.70 for the total scale, 0.60 for the LV subscale, and 0.77 for the FR subscale. These values are comparable to the original English CTI-11 (α = 0.72–0.79) [2] and outperform earlier versions, such as the 30-item CTI (α = 0.58–0.74) [3] and the 18-item CTI (α = 0.73–0.79) [5]. The test–retest reliability (ICC = 0.91) of the CTI-11A is notably higher than typically reported in other validations, such as the Chinese CTI-11, where temporal stability was implied but not quantified [7]. This high ICC suggests that the CTI-11A maintains strong consistency over time in Arabic-speaking populations, potentially due to the rigorous translation process ensuring item clarity.

The CFA for the CTI-11A supported the two-factor structure (LV and FR) similar to those in the English CTI-11, where CFA confirmed the two-factor model, but specific loadings were not detailed [2]. The CTI-11A’s model fit indices (RMSEA = 0.06, CFI = 0.93, TLI = 0.91, SRMR = 0.05) are comparable to other studies, such as the Chinese validation, which reported CFI and TLI values closer to 0.90 [7]. 

The explained variance in the CTI-11A (50.04%; LV = 20.24%, FR = 29.80%) closely mirrors the original CTI-11 (50%) [2], surpassing the 30-item (27%) [3] and 18-item versions (26%) [5], indicating that the CTI-11A retains the enhanced explanatory power of the shorter scale.

In the future, we recommend the introduction of new survey items for the CTI-11A to effectively capture the impact of cultural practices, such as Ramadan and the morning Fajr prayer, which significantly disrupt sleep–wake cycles and eating schedules in Arabic-speaking populations due to early morning prayers and intermittent fasting.

Convergent validity was supported by moderate correlations between the CTI-11A and the JSS (r = 0.40, *p* < 0.001), with the FR subscale (r = 0.36) showing a stronger association than the LV subscale (r = 0.25). This pattern aligns with findings from Kang and colleagues, where FR scores correlated more strongly with sleep quality measures among Chinese nurses, suggesting that circadian flexibility is a critical correlate of sleep outcomes across cultures [7]. Compared to validations in Persian and French, which reported moderate correlations with the Composite Scale of Morningness (CSM) (LV: moderate, FR: weak), the CTI-11A’s correlations with JSS are slightly weaker, possibly due to differences in sleep assessment tools. The heterotrait–monotrait ratio (HTMT = 0.35) confirms discriminant validity, consistent with prior studies [2], indicating that LV and FR measure distinct constructs. Measurement invariance across gender, established through multigroup CFA (ΔCFI ≤ 0.01, ΔRMSEA ≤ 0.015, ΔSRMR ≤ 0.03), mirrors findings in Chinese samples [7], reinforcing the CTI-11A’s applicability across genders.

The LCA identified two latent classes (Class 1: 52%, Class 2: 48%) based on LV, FR, and JSS, with moderate entropy (0.64), indicating distinct but not sharply separated profiles. This contrasts with Kang and colleagues, who identified four latent classes among Chinese nurses (high response: 14.4%, high flexible: 20.1%, high languid: 51.1%, low response: 14.4%), with the high languid class showing higher presenteeism and lower work-related flow compared to the high flexible class [7]. The CTI-11A’s two-class model may reflect a simpler dichotomy in the Bahraini sample, possibly due to cultural homogeneity or a smaller sample size (N = 468 vs. 568 in the paper by Kang and colleagues [7]). Class 1 likely represents vigorous, flexible individuals with better sleep quality (lower JSS scores), while Class 2 may include languid, rigid individuals with poorer sleep quality (higher JSS scores). This aligns with previous findings that languid types experience more sleep disturbances and work errors [7], and with Jafari Roodbandi and colleagues who noted that languid shift nurses suffered more sleepiness and errors [15].

The moderate entropy (0.64) in the CTI-11A’s LCA suggests less distinct class separation compared to what might be expected in studies with higher entropy (not reported in [7], potentially limiting the precision of profile differentiation. However, the two-class structure is consistent with theoretical expectations of circadian variability [4], where vigorous/flexible individuals adapt better to shiftwork than languid/rigid ones. Compared to Baehr and colleagues, which linked circadian amplitude to morningness–eveningness [16], the CTI-11A’s classes provide a more nuanced view by integrating sleep quality (measured by JSS), highlighting the interplay between circadian type and sleep outcomes.

The two latent classes suggest distinct sleep profiles with practical implications for shiftwork management in Arabic-speaking populations. Class 1 individuals, likely vigorous and flexible, may require minimal intervention, focusing on maintaining adaptive sleep habits, similar to the highly flexible class, who exhibited better work-related flow [7]. Class 2 individuals, potentially languid and rigid, could benefit from targeted interventions like sleep hygiene education, flexible scheduling, or circadian alignment strategies (e.g., light therapy) for languid shiftworkers [15]. The LV-JSS correlation (r = 0.25) supports the idea that vigorous individuals have better sleep quality, while the stronger FR-JSS correlation (r = 0.36) indicates that flexibility enhances adaptability to irregular schedules, reducing sleep disturbances. These findings align with Tucker and colleagues, who noted that flexible circadian types tolerate shiftwork better, and extend their implications to Arabic-speaking shiftworkers [6].

The CTI-11A validation used a convenience sample (N = 468) from Bahrain, limiting generalizability across diverse Arabic-speaking populations, unlike the multi-hospital Chinese sample [7]. Self-report measures (CTI-11A, JSS) may introduce biases (e.g., social desirability), a limitation shared with prior CTI studies [2,5]. The moderate LCA entropy (0.64) suggests less distinct class separation compared to potentially higher entropy in four-class models, possibly due to sample size or cultural factors [7]. The suboptimal CFA fit indices (CFI = 0.04, TLI = 0.07) indicate potential item-level issues, less prominent in other validations [7], suggesting a need for item refinement in Arabic contexts.

Future research should validate the CTI-11A in larger, multi-country Arabic samples to enhance generalizability, similar to multi-hospital approach [7]. Incorporating objective measures like actigraphy could mitigate self-report biases [16]. Exploring additional latent classes (e.g., four-class model) and improving CFA fit indices could clarify subgroup differences and strengthen the factor structure. Longitudinal studiesshould examine the CTI-11A’s predictive validity for health outcomes (e.g., fatigue, errors) in Arabic-speaking shiftworkers, particularly in high-shiftwork industries like healthcare [6].

The current study did not explicitly investigate the influence of childcare responsibilities on circadian rhythms and sleep quality among Arabic-speaking shift workers. However, previous research indicates that childcare, particularly during the first year, can significantly disrupt circadian rhythms and sleep patterns due to irregular sleep schedules and increased nighttime awakenings [16]. Recent evidence further supports this, demonstrating that parental involvement in childcare exacerbates sleep challenges for shift workers, including older night shift nurses who report inconsistent sleep timing and prolonged wakefulness affected by family obligations such as childcare. Additionally, during the COVID-19 pandemic, caregivers of young children experienced increased insomnia severity and reduced sleep efficiency associated with childcare disruptions and altered sleep–wake routines, intensifying circadian misalignment [17]. This issue may be particularly pertinent for shift workers, who already contend with circadian misalignment due to non-standard work hours [18]. Therefore, the presence of childcare responsibilities among participants could represent an underappreciated variable influencing the observed results, such as the moderate entropy (0.64) in the latent class analysis, suggesting less distinct separation between the vigorous/flexible and languid/rigid profiles. Future studies should consider incorporating childcare involvement as a covariate to better elucidate its impact on circadian preferences and sleep quality in this population, potentially enhancing the precision of latent class differentiation and the applicability of the CTI-11A in diverse Arabic-speaking contexts.

### 3.1. Strengths and Limitations

#### 3.1.1. Strengths

This study exhibits several significant strengths. First, the rigorous translation and validation process for the Arabic version of the CTI-11A ensured linguistic and cultural equivalence with the original instrument, thereby enhancing its applicability to Arabic-speaking populations. The participation of bilingual medical professionals and sleep researchers, along with a pilot study and cognitive interviews, contributed to the clarity and cultural relevance of the CTI-11A. Second, the study utilized adequate sample size that exceeded the minimum item-to-participant ratio required for factor analysis, providing sufficient statistical power to validate the two-factor structure through CFA and to explore latent circadian profiles via LCA. Third, the comprehensive statistical approach employed, including CFA, multigroup CFA for gender invariance, and correlations with the JSS, supported the psychometric reliability and validity of the CTI-11A, consistent with prior validations in other languages.

#### 3.1.2. Limitations

Despite these strengths, the study has several limitations. The use of convenience sampling through social media platforms for recruitment may have introduced sampling bias toward younger or more tech-savvy individuals, potentially limiting the generalizability of findings to broader Arabic-speaking populations, including older adults or those with limited access to or familiarity with digital platforms. This recruitment method may not fully capture the diverse demographic and socioeconomic characteristics of Arabic-speaking shift workers, particularly in regions with varying levels of technological adoption. Additionally, the reliance on self-report measures (CTI-11A and JSS) introduces potential biases, such as social desirability or recall inaccuracies, which may affect the accuracy of reported circadian preferences and sleep quality. The moderate entropy in the LCA suggests less distinct class separation, possibly influenced by unexamined variables such as childcare responsibilities, which recent studies indicate can significantly disrupt circadian rhythms and sleep patterns, particularly among shift workers. The focus on a Bahraini sample further limits the generalizability to other Arabic-speaking countries with differing cultural or work-related contexts. Future research should address these limitations by incorporating diverse recruitment methods, objective sleep measures (e.g., actigraphy), and multi-country samples to enhance the applicability and robustness of the CTI-11A across varied Arabic-speaking populations.

## 4. Materials and Methods

### 4.1. Translation Process

To ensure linguistic, cultural and psychometric equivalence with the original CTI-11A, the Arabic version of the CTI-11A was developed using a rigorous translation and validation process. Initially written permission was obtained from the original developers of the CTI-11. The translation was carried out following standards for cross-cultural adaptation of self-report measures. This consisted of bilingual medical professionals, fluent in English and Arabic, and experienced in sleep and circadian rhythm research, independently translating the CTI-11 into Arabic. They both also independently commented on the various translations to ensure that a single version could be produced. The diverse versions produced by the translators were then synthesized by expert panel of psychologists and sleep researchers to produce a single reconciled version of the CTI-11 in Arabic. The reconciled version of the CTI-11A was then back-translated into English by two other bilingual medical professionals, who were not informed of the original CTI-11, to ensure conceptual accuracy of the measure. The back-translation was compared to the original CTI-11 in English, and any discrepancies were noted, examined, and resolved by the panel of experts to ensure that the Arabic version of the CTI-11A was true to the content and intent of the original measure.

A pilot study with 20 participants representative of the target population was conducted to determine the clarity, cultural appropriateness, and comprehensibility of the Arabic CTI-11A. Participants completed the Arabic CTI-11A and provided their feedback on their understanding of the items, clarity of the instructions, and overall coherence. Additionally, cognitive interviews were conducted to understand their interpretations of the items and help identify ambiguities. Feedback from the pilot study indicated that the Arabic CTI-11A was well comprehended and culturally appropriate, and did not require any modifications. To minimize possible bias, data from the pilot study were not included in the following analyses. After this pilot study phase, a larger sample was used to evaluate the psychometric performance of the Arabic CTI-11A. The reliability of the Arabic CTI-11A was assessed with internal consistency and test–retest reliability; the validity was obtained with concurrent and convergent measures by comparing the Arabic CTI-11A to previously established sleep-related measures (i.e., the PSQI and the AIS). We follow the same process to evaluate the Arabic CTI-11A as we did with the original English version, which ensured rigorous psychometric evaluation. 

### 4.2. Data Collection

A convenience sample of 468 individuals was obtained via ads on several social media sites, including X (previously Twitter), Facebook, Instagram, and WhatsApp. The research population comprised Arabic-speaking persons aged 18 years and older from Bahrain. Before data collection, the Research Ethics Committee of The Psychiatric Hospital/Government Hospitals in Bahrain evaluated and approved this study (number: 2024-12-364; decision date 5 December 2024). Participation was voluntary, and those who chose to participate provided online informed consent prior to completing the questionnaire, in accordance with the principles set forth in the Helsinki Declaration. Data collection was conducted using an online questionnaire utilizing Google Forms.

No incentives were provided for participation, ensuring that engagement was entirely voluntary and free from external motivation. The screening process was exclusively dedicated to verifying that participants had engaged in shift work within the past month, confirmed through a single eligibility question integrated into the online questionnaire to maintain relevance to the target population of shift workers.

### 4.3. Sample Size and Data Processing

In factor analysis, researchers typically employ the item-to-participant ratio as a criterion for assessing sample size sufficiency, with standard ratios varying from 1:5 to 1:10. In this investigation, we originally employed a cautious strategy, aiming for the minimum suggested ratio of 1:5. Considering that the CTI-11A has 11 questions, this minimal ratio required was 110 participants. To enhance statistical power, ensure robust model estimation, and facilitate multigroup CFA for evaluating measurement invariance across gender, a target sample size of approximately 500 participants was established. This target was determined based on power analysis guidelines for CFA, which recommend a minimum sample size of 200–400 participants to achieve stable parameter estimates and adequate power (0.80) for detecting acceptable model fit with a two-factor model, assuming moderate factor loadings and a significance level of 0.05. The final analytic sample comprised 468 individuals.

To maintain data integrity and avoid duplicate responses to questionnaire items, various procedures were employed. Responses were screened and verified, followed by data cleaning methods to remove any possible duplicate entries. Any questionable or incoherent responses were subjected to data quality assessments to detect random replies or indications of inattentiveness or insufficient effort. One statistic was the participants’ response time. If a participant was deemed a rapid responder, such as indicated by answering all questions in under three minutes, they were assumed to have not thoroughly read or contemplate the questions, likely leading to arbitrary or rushed replies, and were removed from the dataset. Based on our pilot study, which indicated an average completion time of approximately 7 min, we established a minimum threshold of 3 min to exclude “rapid responders”. Responses completed in significantly less time may raise concerns regarding data quality and participant engagement.

Another screening measure was the consistency or uniformity of responses. For example, if a participant repeatedly selected the identical answer option (e.g., continually picking “3” on a Likert scale poll), it was interpreted as a deficiency in attention or effort and such responses were screened out prior to analysis. In total, 12 responses were removed, all of which were duplicates, ensuring the dataset was free of identified errors such as duplicates, rapid responses, and inconsistent answers.

### 4.4. Instruments

#### 4.4.1. Circadian Type Inventory (CTI-11)

The CTI-11 is designed to measure individual differences in circadian types focusing on (1) stability and amplitude of circadian rhythms. The CTI-11 is an 11-item instrument with 2 subscales: Flexible/Rigid (FR, 5 items), which measures the stability of circadian rhythms and adaptability to shift work along with Languid/Vigorous (LV, 6 items), which measures the amplitude of circadian rhythms and tolerance to desynchronization. Respondents rate agreement to items using a Likert-type 5-point scale of 1 (not true at all) to 5 (almost always true). The original English version of the CTI-11 has demonstrated good psychometric properties with Cronbach’s alpha of 0.89 for FR and 0.73 for LV. Because of its exceptional reliability, validity, and utility, the CTI-11 is an important document for researchers and practitioners seeking to better understand circadian preferences and shift work tolerance [1,2,3].

As part of our process for translating the CTI-11A to Arabic, we used the original 11 items and repeated exploratory analyses to verify the subscale structure. All items were retained in the study because they loaded appropriately on their respective factors.

#### 4.4.2. Jenkins Sleep Scale (JSS)

The JSS [19] is a self-report measure that examines sleep problems and disturbances. It is made up of four items: trouble falling to sleep, trouble staying asleep, waking up multiple times each night, and waking up feeling tired and worn out. Participants responded to each item on a scale of 0 (never) to 5 (every day) to reflect how often they experienced each sleep issue over the past month. Higher total scores reflect worse and more disturbed sleep. The JSS has demonstrated adequate reliability and validity across a variety of populations and has been translated into many languages. The reliability of the JSS was translated and validated into a variety of languages including English, Portuguese, Urdu, Turkish, Spanish, German, and Finnish, which were represented with Cronbach’s alpha values ranging from 0.63 to 0.90. For this study the Arabic validated version of the JSS [20] was utilized and reported to demonstrate a Cronbach’s alpha similar to English Version of about 0.80.

### 4.5. Statistical Analyses

The statistical analysis was conducted utilizing R Statistical Foundation. R version 4.4.2 (Pile of Leaves) was released on 31 October 2024. Statistical significance was set at *p* < 0.05.

Internal reliability was assessed using Cronbach’s alpha and McDonald’s omega. Values of 0.70 or more for Cronbach’s alpha and McDonald’s omega were deemed acceptable and satisfactory.

We conducted a confirmatory factor analysis (CFA) to authenticate the factor structure derived from the English-language version of 11 items. Prior to this, we performed an exploratory factor analysis (EFA), which confirmed the two-factor structure without suggesting item deletions.

Confirmatory Factor Analysis (CFA) tests the factor structure of a measure with factor loading and residual variance, where factor loading expresses the strength of the relationship between observable variables and latent factors. We evaluated model fit on several goodness-of-fit metrics. The metrics are comparative fit index (CFI), Tucker–Lewis index (TLI), chi-square statistic, root mean square error of approximation (RMSEA), and standardized root mean square residual (SRMR). A CFI score of 0.95, RMSEA < 0.10, and SRMR < 0.06 is good fit according to recommended values. We also assessed fit with Akaike information criterion (AIC), Bayesian information criterion (BIC) and sample-size-adjusted Bayesian information criterion (SSABIC) scores, where lower is better. The discriminant and convergent validity of the components of the model is evaluated using HTMT and AVE. HTMT cannot be >0.85, and the AVE of all constructs must be >0.50. Using both of these measures enhances validation of the model and indicates a strong match. HTMT evaluates discriminant validity by calculating the ratio of between-trait correlations to within-trait correlations, with values below 0.85 indicating that the constructs are distinct from one another. AVE assesses convergent validity by quantifying the average variance in observed items explained by each latent construct, with values above 0.50 suggesting that the construct accounts for a greater proportion of variance in its items than error.

To assess measurement invariance across gender, multi-group confirmatory factor analysis (MG-CFA) was conducted using a sequential approach to evaluate whether the factor structure, loadings, and intercepts of the CTI-11A were equivalent between male and female participants. Invariance testing proceeded in stages: configural invariance (unconstrained model to confirm the same factor pattern across groups), metric invariance (constraining factor loadings to be equal), and scalar invariance (further constraining item intercepts to be equal). Changes in model fit were evaluated to determine the level of invariance supported, with thresholds including a decrease in comparative fit index (ΔCFI) ≤ 0.01, an increase in root mean square error of approximation (ΔRMSEA) ≤ 0.015, and an increase in standardized root mean square residual (ΔSRMR) ≤ 0.03 indicating acceptable invariance. Nested model comparisons were performed using chi-square difference tests (Δχ^2^), with non-significant results (*p* > 0.05) supporting invariance. The Akaike information criterion (AIC) and Bayesian information criterion (BIC) were also examined, where lower values in constrained models relative to unconstrained ones further indicate invariance without substantial loss of fit.

Convergent validity was assessed using Pearson’s product moment correlation coefficients between the CTI-11A total score, its subscales, and the JSS total score. Significant positive correlations were expected, supporting convergent validity.

Latent Class Analysis (LCA) was performed using the glca R 4.4.2 package to identify distinct profiles based on the manifest items from the LV and FR subscales of the CTI-11A and JSS. The analysis aimed to determine the optimal number of latent classes, with a focus on extracting two classes based on theoretical foundations and preliminary model fit assessments. Model fit was evaluated using several indices, including log-likelihood, Akaike Information Criterion (AIC), Bayesian Information Criterion (BIC), and sample-size-adjusted Bayesian Information Criterion (CAIC), where lower values indicated better-fitting models. Additionally, the entropy value was computed to assess the clarity of class assignments. The G-squared statistic and a bootstrap *p*-value were utilized to evaluate the significance of the model fit, both indicating a strong fit to the data. Marginal prevalence for each latent class was calculated to report the proportion of participants classified into each group, reflecting their distribution across the identified profiles.

## 5. Conclusions

The CTI-11A demonstrates robust psychometric properties, with reliability and validity comparable to prior CTI-11 validations, though model fit requires refinement. The two latent classes highlight diverse sleep profiles, offering practical insights for tailored sleep interventions in Arabic-speaking shiftworkers, consistent with findings from previous studies. This study advances circadian research in Arabic contexts, supporting its application in occupational health while identifying areas for further cross-cultural exploration.

## Figures and Tables

**Table 1:** Descriptive Statistics, Skewness, and Kurtosis for Study Variables (N = 468).

Variable	M	Mdn	SD	IQR	Skewness	Kurtosis
Age	36.18	36.00	10.35	17.00	0.11	−1.14
LV1	3.16	3.00	1.21	2.00	−0.25	−0.74
LV2	3.41	3.00	1.17	1.00	−0.36	−0.61
LV3	3.75	4.00	1.10	2.00	−0.66	−0.25
LV4	3.38	3.00	1.11	1.00	−0.29	−0.52
LV5	3.19	3.00	1.23	2.00	−0.20	−0.85
LV6	3.05	3.00	1.14	2.00	0.03	−0.66
FR1	3.31	3.00	1.26	2.00	−0.26	−0.96
FR2	2.87	3.00	1.45	3.00	0.05	−1.34
FR3	3.20	3.00	1.12	2.00	−0.20	−0.65
FR4	3.00	3.00	1.35	2.00	−0.00	−1.13
FR5	3.08	3.00	1.34	2.00	−0.13	−1.09
LV (total)	19.94	20.00	3.91	6.00	−0.15	−0.14
FR (total)	15.46	16.00	4.75	7.00	−0.04	−0.74
CTI-11A (total)	35.40	35.00	6.61	9.00	0.07	−0.18
JSS (total)	5.76	5.00	3.48	5.00	0.43	−0.47

Notes: M = Mean; Mdn = Median; SD = Standard Deviation; IQR = Interquartile Range; Age = Age in years; LV 1–6 = Item 1–6 of Languid/Vigorous (LV) subscale of CTI-11; FR1-5 = Item 1–5 of Flexible/Rigid (FR) subscale of CTI-11; LV3 = Item 3 of LV subscale of CTI-11; LV (total) = Total score for LV subscale of CTI-11; FR (total) = Total score for FR subscale of CTI-11; CTI-11 (total) = Total score for Circadian Type Inventory (CTI-11); JSS (total) = Total score for Jenkins Sleep Scale.

**Table 2:** Confirmatory factor analysis of the CTI-11A.

Factor	Indicator	Estimate	*p*-Value	Residual Covariance	*p*-Value
LV	LV1	0.55	<0.001	0.70	<0.001
	LV2	0.47	<0.001	0.78	<0.001
	LV3	0.32	<0.001	0.90	<0.001
	LV4	0.60	<0.001	0.64	<0.001
	LV5	0.58	<0.001	0.67	<0.001
	LV6	0.05	<0.001	1.00	0.36
FR	FR1	0.56	<0.001	0.69	<0.001
	FR2	0.79	<0.001	0.38	<0.001
	FR3	0.41	<0.001	0.83	<0.001
	FR4	0.74	<0.001	0.45	<0.001
	FR5	0.66	<0.001	0.57	<0.001

Notes: LV = Languid/Vigorous subscale of CTI-11A; FR1 = Flexible/Rigid (FR) of CTI-11A. Standardized estimates using Maximum Likelihood Estimator (MLE). Model Fit: Test for Exact Fit: χ^2^ = 103.90; df = 43; *p* < 0.001; Fit Measures: RMSEA = 0.06 [90% CI: 0.04, 0.07], CFI = 0.93, TLI = 0.91, SRMR = 0.05); AIC = 15,898.17; BIC = 16,039.22; SRMR = 0.04. Reliability analyses of the entire CTI-11A = 0.72 for α and ω; for LV, α and ω were 0.70; for FR, α and ω were 0.77. Heterotrait–monotrait (HTMT) ratio: 0.35; Mardia’s coefficients: Skewness: Coefficient = 5.50; χ^2^ = 429.27; df = 286; *p* < 0.001; Kurtosis: Coefficient = 149.06; z = 3.88; *p* < 0.001.

**Table 3:** Correlation matrix of CTI-11A and JSS.

Variable	LV	FR	CTI-11	JSS
LV	—			
FR	0.16 *	—		
CTI-11A	0.70 *	0.81 *	—	
JSS	0.25 *	0.36 *	0.40 *	—

Notes: LV = Languid/Vigorous subscale of CTI-11A; FR1 = Flexible/Rigid (FR) of CTI-11A; JSS = Jenkins Sleep Scale. * *p* < 0.001.

**Table 4:** Latent class analysis of CTI-11A factors and JSS.

Manifest Items	LV, FR, JSS
Classes/Profiles	2
Log-likelihood	−3796.23
AIC	7822.45
CAIC	8414.53
BIC	8299.53
Entropy	0.64
Degrees of Freedom (df)	352
G^2^	1972.66
*p*-value	<0.001
Marginal Prevalence for Latent Class	Probability
Class 1	52%
Class 2	48%

Notes: LV = Languid/Vigorous subscale of CTI-11; FR1 = Flexible/Rigid (FR) of CTI-11; JSS = Jenkins Sleep Scale.

## Data Availability

The data are not publicly available due to privacy or ethical restrictions. The data presented in this study are available upon request from the corresponding author.
